# P-1148. Patient Harm Events: Hospital-Onset Bacteremia vs CMS-Reportable Events

**DOI:** 10.1093/ofid/ofaf695.1342

**Published:** 2026-01-11

**Authors:** Heather Young, Carolyn Valdez, Timothy C Jenkins

**Affiliations:** Denver Health, Denver, CO; Denver Health, Denver, CO; Denver Health, Denver, CO

## Abstract

**Background:**

CMS requires hospitals to report rates of CLABSI, CAUTI, SSI after colon surgery and abdominal hysterectomy, hospital-onset MRSA bacteremia, and hospital-onset *C. difficile* infection (HO-CDI) to NHSN. With any process that requires manual adjudication, discrepancies in data accuracy exist. To identify patient harm and to maximize data accuracy, a laboratory-reported hospital-onset bacteremia (HOB) event has been proposed as an alternative to certain CMS-reportable events.

The goal of this study is to compare the performance of the proposed HOB metric with traditional CMS metrics for the identification and prognosis of HAI.

**Table 1.**
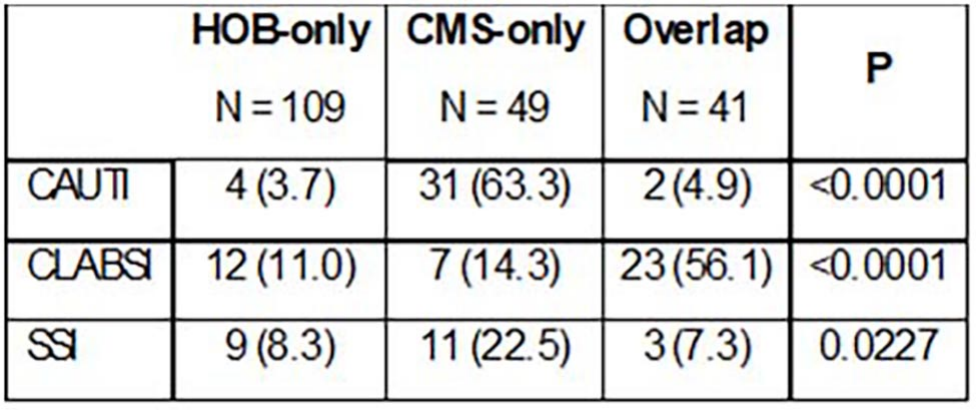
Source of Infection and HAI Group

**Methods:**

This is a retrospective cohort study of patients hospitalized between 1/1/22 and 12/31/23 at a 500-bed academic safety net hospital in Denver, CO. Patients >18 years old with a positive blood culture on hospital day 4 or greater OR a CMS-reportable HAI were eligible for inclusion; those with HO-CDI were excluded. Patients were categorized into 3 mutually exclusive groups: HOB-only if the HAI was not reportable to CMS; CMS-only if the HAI did not involve bacteremia; or Overlap if the HAI involved bacteremia and was reported to CMS.

**Results:**

Overall, 199 HAIs were identified in 185 patients by HOB or a current CMS metric. There were 109 HOB-only events (54.8%) in 97 patients, 49 CMS-only events (24.6%) in 49 patients, and 41 Overlap events (20.6%) in 39 patients. Age, gender, race, ethnicity, and preferred language did not differ between the 3 groups. The 6-month mortality was not different between the three groups (28.4% vs 20.4% vs 24.4% respectively, P=0.553).

While CLABSI was most prevalent in the Overlap group, CAUTI and SSI were more strongly represented in the CMS-only group than in the HOB or Overlap groups (Table 1).

Infections due to Gram negative organisms were more prevalent in the CMS-only than in the HOB-only or Overlap groups (69.4% vs 41.3% vs 14.6%, P< 0.0001) as were polymicrobial infections (32.7% vs 11.0% vs 4.9%, P=0.0003).

**Conclusion:**

A change from current CMS-reported HAI events to HOB would lead to an overall increased number of identified patient harm events; however, CAUTI and SSI events would likely decrease. Care should be taken to ensure that the SSI events captured by HOB reflect emergent, urgent, and elective surgery types.

**Disclosures:**

All Authors: No reported disclosures

